# Evaluation of Stress Scores of Healthy Adult Cats during Barometric Whole-Body Plethysmography and Its Correlation with Measurement Parameters

**DOI:** 10.3390/ani14152249

**Published:** 2024-08-02

**Authors:** Petra Benz, Yury Zablotski, Bianka Schulz

**Affiliations:** Small Animal Clinic, Centre for Clinical Veterinary Medicine, Ludwig Maximilian University of Munich, 80539 Munich, Germany; y.zablotski@med.vetmed.uni-muenchen.de (Y.Z.); bianka.schulz@lmu.de (B.S.)

**Keywords:** feline stress score, cat behaviour, feline asthma, lung function testing, non-invasive

## Abstract

**Simple Summary:**

The stress load of cats undergoing lung function diagnostics using barometric whole-body plethysmography has not yet been investigated. In this study, a feline stress score was used to determine the stress level of healthy cats over a 30 min time period during plethysmographic measurement; in addition, the correlation with measured parameters was evaluated. The stress levels of the majority of the 48 cats studied were increased at the beginning of measurements and decreased significantly over time. There was a significant correlation between most measurement parameters and the total stress score. Although the barometric whole-body plethysmography is considered to be a particularly gentle method, most cats initially experienced moderate stress. The stress level must be taken into account when interpreting the measured parameters.

**Abstract:**

Barometric whole-body plethysmography (BWBP) is considered to be a particularly gentle method of assessing lung function in cats. However, there have been no studies to date investigating the stress experienced by cats during measurements. The prospective study included 48 healthy adult cats. Each cat was measured in the plethysmographic chamber for a total of 30 min and stress levels were determined every 10 min using a stress ethogram. At the beginning of measurements, 75% of cats were assessed as tense. Over the three time periods, a significant (*p* < 0.001) reduction in the total stress score was observed. In addition, all measurement parameters correlated significantly with the stress score, with the exception of enhanced pause and tidal volume. It can therefore be assumed that cats will initially experience stress during examination in the plethysmographic chamber, but stress will decrease significantly over time. As the stress level correlates with many measurement parameters, this should be taken into account when interpreting the results.

## 1. Introduction

Domestic cats are popular pets all over the world [1,2]. With 1–5% of the cat population suffering from feline lower airway disease (FLAD) [3,4], veterinary visits are often unavoidable. Visits to the vet are considered stressful for animals because of the unfamiliar surroundings, smells, noises, and people [5,6,7]. Stress in cats manifests in vocalisations, posture, ears, whiskers, and eyes [5,8,9,10]. However, stress is not only visible on the outside, it also has an effect on vital parameters such as blood pressure, rectal temperature, heart rate, and respiratory rate [11,12,13]. Particular attention should be paid to the effect of stress on respiration. Breathing is primarily regulated without active control by the respiratory centre [14,15]. The respiratory centre responds to metabolic changes in CO_2_, O_2_, and pH by adjusting respiratory rate and depth of breathing (tidal volume) [16,17], which also affects lung function. However, breathing and lung function can also be influenced by emotions such as fear, sadness, and happiness, as several studies in humans have shown [14,15,18,19,20,21]. Emotional changes in breathing can be unconscious, but can also be voluntary [17]. Emotions affect respiratory performance through a complex interaction among the brainstem, cerebral cortex, and limbic system [14,17]. A special role in processing emotions is played by one part of the limbic system, the amygdala [14,17,22]. There is evidence that the electrical stimulation of the amygdala can induce specific symptoms of anxiety [14]. This could be shown in a study in cats, in that the stimulation of the amygdala resulted in an increase in respiratory rate [23]. Lung function diagnostics, which can be helpful to assess airflow limitation in cats with FLAD, often involve manipulation and sometimes even anaesthesia, which is particularly stressful for the cat. Barometric whole-body plethysmography (BWBP) represents an option for lung function testing that can be performed without manipulation and restraint while the patient is awake. Therefore, it seems very well accepted by cats [24]. However, the unfamiliar environment and situation, in addition to the sounds and smells in the plethysmographic chamber, are also potential stress triggers. Negative emotions, in turn, affect the respiratory centre via the limbic system, and could thus influence lung function parameters.

The aim of this study was to investigate the stress level of cats in the plethysmographic chamber, to assess its course during acclimatisation time, and to correlate the stress level with the measured parameters. The authors hypothesise that the unfamiliar situation in the plethysmographic chamber influences the stress level of cats, and as a consequence, the measurement parameters of BWBP.

## 2. Materials and Methods

### 2.1. Ethical Approval

The Ethics Committee of the LMU Munich Centre for Clinical Veterinary Medicine approved all study procedures (211-07-04-2020). Owner consent was given for all participants. 

### 2.2. Study Population

The prospective observational study involved 48 cats owned by staff of the teaching hospital, students, or cats that were presented to the clinic for annual health checks. The study was performed from May 2020 to September 2021. Only clinically healthy cats at least one year of age with a normal body condition score (BCS) (range 3/9 to 6/9) were included in the study. All cats were in the plethysmographic chamber for the first time. The study population has already been the subject of another study evaluating the influence of acclimatisation time on BWBP parameters [25].

### 2.3. Study Design

All cats were examined clinically prior to the BWBP measurement. The cats spent 30 min in the plethysmographic chamber and lung function measurements were recorded during that time period. For evaluation, the measurement period was divided into three units of 10 min each (T1 = 0–10 min, T2 = 10–20 min, T3 = 20–30 min). During the 30 min of measurement in the plethysmographic chamber, the cats were evaluated for stress in 10 min sections using a stress score. The investigator was present during the entire measurement period to assess the behaviours of the animals at all times. 

### 2.4. Barometric Whole-Body Plethysmography

**Figure 1:**
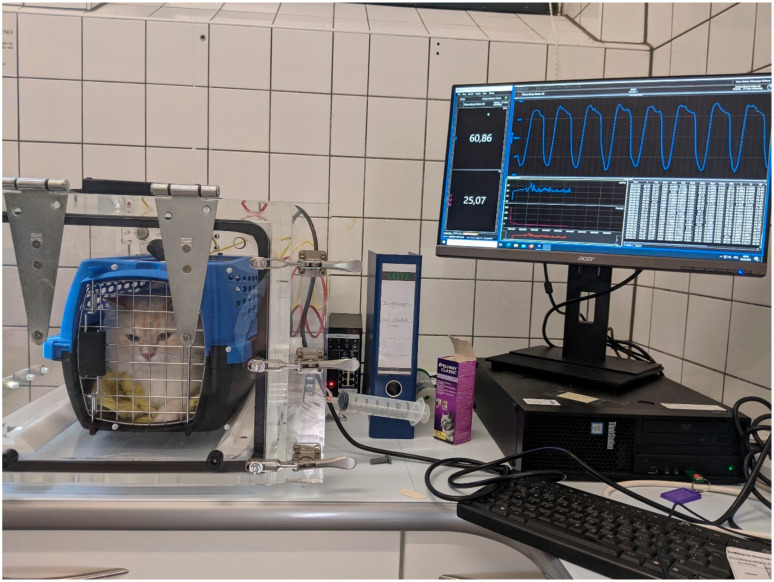
Image of a cat in the plethysmographic chamber. The lung function diagnostic recordings are recorded with the software program. © Small Animal Clinic, LMU Munich.

BWBP was performed as described before [25]. Briefly, the cats were placed in a transparent chamber inside a standardised transport box (Figure 1). The transport box was sprayed with two puffs of Feliway^®^ (Ceva Tiergesundheit GmbH, Düsseldorf, Germany), a cat calming drug, before placing the cat into it. To ensure the continuous ventilation of the chamber with fresh air, bias flow was used (Buxco^®^ Multi-function Bias Flow, Data Science International (DSI), New Brighton, MN, USA). Air flow was provided by sieve pneumotachographs. The pressure changes within the chamber were recorded using a flow transducer (Halcyon™ pneumotach, Data Science International (DSI), New Brighton, MN, USA), and with the help of the preamplifier (Buxco^®^ QT Digital Preamplifier, Data Science International (DSI), New Brighton, MN, USA), measurements were amplified, digitised and forwarded to the computer software program v2.9.0 (Buxco^®^ FinePoint Small Animal Whole Body Plethysmograph, Data Science International (DSI), New Brighton, MN, USA) for analysis.

The measured and calculated parameters are listed in Table 1.

### 2.5. Assessment of Stress Level

For the assessment of the cats’ stress level during plethysmographic measurement, an adapted Kessler and Turner cat-stress-score (CSS) was used [26]. The assessment was non-invasive and did not affect the cats’ behaviour. The assessment of the stress level was always performed by the same observer, who sat quietly in front of the plethysmographic chamber for the entire 30 min period. Each assessment was made for a 10 min observation period. Thus, three different stress scores were documented for each cat over three different time periods. The stress ethogram consisted of seven categories. These categories included 1 “relaxed”; 2 “alert relaxed”; 3 “tense”; 4 “strongly tense”; 5 “stiff”; 6 “anxious”; and 7 “panicky”. The behavioural elements used to assess the stress categories are shown in Table 2.

### 2.6. Statistical Analysis

Statistical analysis was performed using SPSS version 28.0.0 software (IBM Corp, Armonk, NY, USA). The Shapiro–Wilk test was used to test for the normal distribution of all data. Since most of the data were not normally distributed, and due to the presence of repeated measures, the Friedman test was applied to assess the evolution of the CSS over the three time periods and to compare the BWBP measurement parameters of the three measurement units. All time points were compared among each other using the Dunn–Bonferroni post hoc test. The Bonferroni *p*-value correction for multiple comparisons was applied to the Friedman test results in order to reduce the Type 1 Error. Spearman correlation coefficient *r* was used to examine the correlation between CSS and BWBP measurement parameters over the three time periods. Table 3 shows the interpretation of the Spearman correlation coefficient *r*. The level of significance was set at *p* < 0.05 for all tests.

## 3. Results

### 3.1. Study Population 

All 48 cats entering the study could be included. The group consisted of 26 males (24 neutered) and 22 females (20 spayed), with a median age of 4.39 ± 4.38 years (range 1–16 years) and a median BMI of 4/9 (range from 3/9 to 6/9). Breeds included European Shorthair (22), Norwegian Forest Cat (6), Siberian Forest Cat (2), Maine Coon (2), British Longhair (1), British Shorthair (3), Birman (4), Bengal (1), British Shorthair mix (1), Norwegian Forest mix (1), Birman ×Persian mix (3), and Bengal ×British Shorthair mix (2). 

### 3.2. Barometric Whole-Body Plethysmography

BWBP was well tolerated by all cats. The results of the BWBP measurements have been published as part of a different study [25].

### 3.3. Cat-Stress-Score

**Figure 2:**
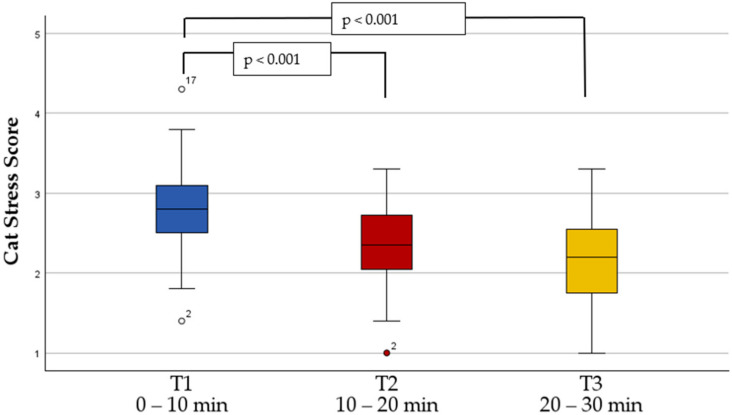
Development of the cat stress score over the three time periods, tested with Friedman test and Dunn–Bonferroni post hoc test. The brackets indicate statistically significant changes (*p* < 0.001). Outliers are indicated by circles, the corresponding number identifies the respective cat.

The development of the CSS over all three time periods is shown in Figure 2. Out of the 48 cats, 3 cats were assessed as strongly tense at the beginning of the study, 36 cats were tense, 8 cats were alert relaxed, and 1 cat was relaxed. Between time periods T1 and T2 and T1 and T3, CSS decreased significantly (*p* < 0.001); between T2 and T3, the decrease was not significant (*p* = 0.109). Table 4 shows the absolute and percentage distribution of cats in the seven CSS categories over the three time points.

### 3.4. Correlation between BWBP Parameters and Cat-Stress-Score

The results of the correlation between the CSS and the BWBP parameters for all three time points are presented in Table 5. Respiratory rate (RR), expiratory time (Te), minute volume per body weight (MV/BW), peak inspiratory flow per body weight (PIF/BW), peak expiratory flow per body weight (PEF/BW), and relaxation time (Tr) correlated moderately with CSS during T1. MV/BW, PEF/BW, and PIF/BW also showed a moderate correlation with CSS at T2 and T3. The parameters pause (PAU) and inspiration time (Ti) were only weakly correlated with the CSS during the whole measuring period. No significant correlation was found between the enhanced pause (Penh) and tidal volume per body weight (TV/BW) and the CSS.

## 4. Discussion

To the authors’ knowledge, this is the first study evaluating the stress score of cats during plethysmographic measurements. The study showed that the majority of cats were tense at the beginning of the measurement, but the stress level decreased significantly over time. In addition, it could be shown that RR, MV/BW, PIF/BW, PEF/BW, Ti, and Te correlated significantly with CSS. 

Most cats react with stress to new environments, smells, unfamiliar faces, and sounds or when they are separated from their owners and locked in a cage [5,6,7,9,11,27,28,29]. Most of these factors apply during transportation and at the veterinarian’s office. Thus, it was not surprising that the majority of cats in this study were assessed as being tense at the beginning of the plethysmographic measurement. Consistent with findings in other studies, the tense behaviour of the cats was expressed as a stiff posture, crouching, a protected belly, a twitching tail held close to the body, and open eyes [5,9,26,29,30,31]. Two study participants were found alert and relaxed (category 2) at the beginning of measurements, but three were even strongly tense (category 4). How a cat reacts to unfamiliar situations is individual and depends on genetics, socialisation, and previous experience [5,6,9,32,33]. Since all the cats in the present study were adult, well socialised, healthy, and had been in a transport box before, there were no cats during the entire measurement period that showed an even higher level of stress expressed as being stiff (category 5), anxious (category 6), or even panicky (category 7). BWBP is considered a non-manipulative and non-invasive method that has been used in studies in cats before to assess lung function without the description of severe stress-related reactions [24,34,35,36]. Therefore, a high stress level in the participating cats was not expected. Another advantage of our study population was that none of the cats had been placed in a transport cage for the first time when the plethysmographic measurements were taken. Cage confinement is initially stressful for many cats because it is a restriction of their normal behaviour [9]. Therefore, it is important that cats are already accustomed to a transport box when BWBP is planned as part of a diagnostic work-up. In addition, Feliway^®^, which has been shown to reduce stress levels in cats during veterinary examinations [37], was used in the present study. It should thus be noted that the stress level of the cats may have been higher during the measurements if Feliway^®^ had not been used. The mean stress level of individual cats decreased by no more than one level per unit of time. No further significant reduction in stress level was observed between T2 and T3. It can be assumed that cats slowly become accustomed to the situation in the chamber while BWBP is performed, but are restricted in their movement and cannot avoid potential stressors (e.g., noise, no hiding places, odours); therefore, the stress potentially remains at the same level after first acclimatisation and does not decrease further. In some cases, the stress scores increased again in the third phase. This may be due to cats becoming more restless and impatient due to the behavioural restrictions. This especially seemed to affect younger and more active cats. Unfortunately, this hypothesis could not be confirmed by a statistical analysis in the present study due to the small number of cats (3) that showed an increase in stress level in T3. The cat population in this study was in the plethysmographic chamber for the first time. Repeated measurements may acclimate the cats to the situation and further reduce stress levels. However, further studies are needed to prove this hypothesis.

It is important to keep in mind that only healthy cats were examined in the present study. It must be taken into account that cats with respiratory diseases are additionally stressed by their underlying disease. Therefore, a higher stress level is generally to be expected in sick cats.

Another important aspect of the study was to find out whether the BWBP parameters were influenced by the CSS during the measurement sessions. When measurements were started, the respiratory rate (RR) was already significantly increased above the range considered physiological in healthy cats (data is shown in Table S2) [38]. It is well known that an unfamiliar environment can trigger fear in cats via the stimulation of the amygdala, leading to an increase in RR [23]. In the present study, the RR correlated moderately and significantly with the CSS during T1. Similarly, studies in humans described a positive correlation between RR and anxiety scores [39]. Later, during the measurement period (T2–T3), only a weak correlation between CSS and RR was present. While the RR only decreased from 65 to 57 breaths/minute from T1–T3 and thus never reached a normal range, whilst the CSS, on the other hand, continued to decrease significantly to the relaxed or alert state, because posture and other behaviour parameters normalised. A reason for the permanently elevated RR could be persistent mild stress. It has been shown that most cats presented to veterinary practices show permanently elevated RR above the reference range [12],;therefore, normal values can probably not be expected during examinations in the plethysmographic chamber. During T1, there was a significant negative correlation of the parameters inspiratory time (Ti) and expiratory time (Te) with the CSS. The negative correlation can be explained by the fact that a reduction in stress leads to a prolongation of the in- and expiratory phases. The results of the present study are consistent with those of human studies in that a decrease in Ti and Te was observed in healthy subjects during a stressful phase [21], and a negative correlation was observed between expiratory time and anxiety scores [40]. 

In the present study, the measurement parameter minute ventilation per body weight (MV/BW) correlated significantly with CSS over all three time periods. It is well known from human studies that MV/BW is influenced by stress and anxiety [15,41]. Hyperventilation in stressful situations may be an explanation for the increase in MV. As a consequence of acclimatisation, in the present study, CSS and RR decreased during the measurements, leading to a reduction in MV. 

Particularly interesting in this study is that the peak expiratory flow per body weight (PEF/BW) correlated significantly with CSS at all three time periods, and peak inspiratory flow per body weight (PIF/BW) correlated at T1. Both parameters have been associated with upper and lower airway obstruction in dogs [42,43] and cats [44] in previous studies. It is known from studies in people that stress can induce bronchoconstriction, particularly in patients with asthma [45]. Two human studies using BWBP and forced oscillation technique were able to show that different emotions can lead to increased airway resistance, even in healthy people [46,47]. Thus, mild-to-moderate airway obstruction induced by the initially higher stress level may also have occurred in the clinically healthy cats in the present study. As the CSS decreases, bronchoconstriction potentially decreases as well. This, in turn, leads to a change in PIF/BW and PEF/BW, explaining the correlation with CSS. The correlation between PIF/BW and CSS during T2 and T3 was still moderate, but no longer significant. At T1, there was a negative significant correlation between the relaxation time (Tr) and CSS. Tr is the time point at which 65% of the tidal volume has been expired. At T1, the CSS correlated significantly with the RR, as both were significantly increased. With a higher RR, Tr occurs more quickly. A higher CSS associated with hyperventilation therefore leads to a lower Tr, which explains the negative correlation during T1. 

The calculated parameter “enhanced pause” (Penh) has been discussed as an indicator of bronchoconstriction in cats [34,35,36,48]. Increased Penh values can be an indication of airway obstruction in mice [49]. In agreement with other studies, which consider Penh to be a stable parameter not influenced by age, weight, or sex [50,51], no significant correlation with CSS was found at any time point in the present study. Therefore, according to the results of the present study, Penh is not expected to be affected by stress.

This study has several limitations. It is known that a variety of different emotions can induce different breathing patterns in humans. However, it is difficult to distinguish between different emotions in cats; therefore, only stress levels were considered in this study. It is also important to note that a cat’s breathing pattern under stress may differ from that of a human under stress. It is also known that cats express stress levels in different ways, often vaguely [5], making interpretation difficult. In addition, the fact that waiting times before measurement in the chamber could also affect stress levels [9] could not be taken into account in this study.

Since cats often experience great stress when separated from their owners [10,32], future BWBP measurements could include the owner in this diagnostic procedure to further reduce the cat’s stress level. For future studies, a presetting evaluating this as a potential influencing factor would be advisable. The authors suggest that short waiting times, familiarisation with the plethysmographic chamber before measurement, and the presence of the owner may further reduce stress levels, but further research is needed to prove this hypothesis. In addition, some measurement parameters are influenced by humidity and temperature in cats [50]. In the current study, however, the plethysmographic chamber was not preheated, which could additionally influence the measured parameters. However, both temperature and humidity were not subject to major fluctuations.

## 5. Conclusions

Although BWBP is considered a non-invasive diagnostic tool, healthy cats are initially stressed by the unfamiliar situation and environment. However, the CSS decreases significantly over the course of the measurements. Most of the measured parameters correlated with CSS, suggesting the usefulness of sufficient acclimatisation time before measurement. Future studies should therefore take the stress level into account when interpreting feline BWBP measurement parameters. 

## Figures and Tables

**Table 1:** BWBP parameters with units and description.

BWBP Parameter	Unit	Description
RR	Breaths/min	Respiratory rate
Ti	s	Inspiratory time
Te	s	Expiratory time
TV/BW	mL/kg	Tidal volume per body weight
MV/BW	mL/min/kg	Minute volume per body weight
PIF/BW	mL/s/kg	Peak inspiratory flow per body weight
PEF/BW	mL/s/kg	Peak expiratory flow per body weight
Tr	ms	Relaxation time; time at which 65% of tidal volume is exhaled
PAU	unitless	Pause, [(Te/Tr) − 1]
Penh	unitless	Enhanced pause, [((Te/Tr) −1) × (PEF/PIF)]

min: minute, s: second, mL: millilitre, kg: kilogram, ms: millisecond.

**Table 2:** Cat-stress-score adapted from Kessler and Turner [26].

Indicator	Behavioural Element	Category
Abdomen	1 = exposed, visible	1–7
	2 = sometimes unprotected	
	3 = protected, not visible	
	4 = protected, not visible	
	5 = protected, not visible	
	6 = protected, not visible	
	7 = protected, not visible	
Breathing	1 = slowly	
	2 = slowly	
	3 = slowly	
	4 = slowly	
	5 = slow to fast	
	6 = fast	
	7 = fast	
Legs	1 = stretched out (front- and hindlegs)	
	2 = underlaid, hind legs stretched out	
	3 = angled	
	4 = angled	
	5 = angled, crouched	
	6 = angled, crouched	
	7 = buckled	
Tail	1 = stretched out, loosely wrapped around body	
	2 = loosely wrapped around body, raised, loosely downward	
	3 = possibly twitching, held to body, or arching backwards	
	4 = close to body, tensed held down, twitching, beating	
	5 = close to body, held forward in an arc	
	6 = close to body, low to the ground, motionless	
	7 = close to body	
Head	1 = chin or cheek resting on the ground	
	2 = propped up or held against the body, slight movements	
	3 = retracted (above the body), smaller movements	
	4 = retracted (above the body), pressed against the body, little or no movement	
	5 = retracted between the shoulders, absent, or weak movement	
	6 = retracted between the shoulders, close to the body, facing away from danger	
	7 = below the body, motionless	
Eyes	1 = closed, half open, slow blink	
	2 = closed, half open, open	
	3 = open (normal)	
	4 = wide open, tightly closed	
	5 = wide open	
	6 = torn up	
	7 = torn up	
Pupils	1 = normal	
	2 = normal	
	3 = normal	
	4 = partially dilated	
	5 = dilated	
	6 = fully delated	
	7 = fully delated	
Ears	1 = normal (sideways or slightly backwards)	
	2 = turned outward or backward, not applied	
	3 = attentively forward	
	4 = attentively forward	
	5 = partially flattened	
	6 = fully flattened	
	7 = facing backwards and fully flattened	
Whiskers	1 = lateral	
	2 = lateral or forward	
	3 = lateral or forward	
	4 = forward	
	5 = forward	
	6 = back	
	7 = back	
Vocalisation	1 = none/quiet	
	2 = none/quiet	
	3 = meow	
	4 = plaintive meow	
	5 = plaintive meow/growling	
	6 = plaintive meow/growling	
	7 = plaintive meow/growling	
Activity	1 = sleeping/resting	
	2 = resting, playing	
	3 = resting, alert, actively prowling	
	4 = cramped sleeping, alert, trying to escape	
	5 = alert, trying to escape	
	6 = creeping, motionless alert	
	7 = motionless alert	
Total cat-stress-score	=Average of all twelve behavioural elements	

**Table 3:** Interpretation of the Spearman correlation coefficient *r*.

Correlation Coefficient *r*	Categorisation
**0.0 < 0.1**	Very weak
**0.1 < 0.3**	Weak
**0.3 < 0.5**	Moderate
**0.5 < 0.7**	Strong
**0.7 < 1.0**	Very strong

**Table 4:** Absolute and percentage distribution of cats assigned to the seven categories of the cat-stress-score.

Category	Time Periods		
	T1 *n* = 48	T2 *n* = 48	T3 *n* = 48
Relaxed	1 (2.1%)	2 (4.2%)	2 (4.2%)
Alert relaxed	8 (16.7%)	29 (60.4%)	32 (66.7%)
Tense	36 (75.0%)	17 (35.4%)	14 (29.2%)
Strongly tense	3 (6.3%)	-	-
Stiff	-	-	-
Anxious	-	-	-
Panicky	-	-	-

**Table 5:** Correlation coefficients *r* between measurement parameters and cat-stress-score at time periods T1, T2, and T3.

BWBP Parameter	Time Periods		
	T1	T2	T3
RR	**0.324**	0.110	0.153
Ti	**−0.297**	−0.081	−0.135
Te	**−0.325**	−0.122	−0.170
TV/BW	−0.176	0.022	−0.011
MV/BW	**0.465**	**0.350**	**0.376**
PIF/BW	**0.372**	0.258	0.249
PEF/BW	**0.481**	**0.351**	**0.356**
Tr	**−0.303**	−0.121	−0.151
PAU	−0.230	−0.074	−0.222
Penh	0.048	0.182	0.047

Values in bold indicate *p* < 0.05.

## Data Availability

The original contributions presented in the study are included in the article/Supplementary Materials, further inquiries can be directed to the corresponding author/s.

## Supplementary Materials

The following supporting information can be downloaded at: https://www.mdpi.com/article/10.3390/ani14152249/s1, Table S1: Data of all 48 cats included in the study; Table S2: Mean values of all BWBP parameters for each cat and each time period.
